# An Atypical Presentation of Pancreatitis Secondary to a Ventriculoperitoneal Shunt

**DOI:** 10.7759/cureus.54160

**Published:** 2024-02-14

**Authors:** Tasciana T Gordon, Katherine Goodall, Joanne Dale

**Affiliations:** 1 General Surgery, Mater Hospital, Brisbane, AUS

**Keywords:** ventricular catheter, hydrocephalus, atypical pancreatitis, pancreatitis, ventriculoperitoneal shunt complications, ventriculoperitoneal shunt

## Abstract

Ventriculoperitoneal (VP) shunts are catheters inserted to drain excess cerebrospinal fluid (CSF) when there is an obstruction in the normal outflow or a decreased absorption of the fluid leading to hydrocephalus. Recognised complications of placement of the distal catheter are malposition, obstruction, pseudocysts and infection. Here, we present a case of a 23-year-old female with acute pancreatitis following the placement of a VP shunt in the lesser sac. The patient originally had a VP shunt placed in infancy for congenital hydrocephalus with one revision at four years old. She presented with a three-day history of worsening epigastric pain with an associated lipase of 3030 (10-60IU), CRP 157 (<5mg/L) and normal liver function tests. A CT scan showed acute pancreatitis with an associated collection within the lesser sac extending to the greater omentum. This was due to the malposition of the VP shunt after a recent revision surgery. It was managed with a diagnostic laparoscopy, washout and shunt externalisation. This is an atypical presentation of acute pancreatitis secondary to a VP shunt. A high index of suspicion is needed for diagnosis. Management of both pancreatitis and VP shunt complications need to be considered.

## Introduction

Pancreatitis is an inflammatory disorder of the pancreas. Common causes include gallstones, alcohol, hyperlipidaemia and autoimmune pancreatitis [1,2]. The diagnosis of pancreatitis is typically stipulated by the presence of two of the three following criteria: upper abdominal pain, elevated lipase (three or more times the upper limit of normal) and radiological findings consistent with pancreatitis [1,2]. Classification scores such as Ranson criteria are utilised to determine the severity of pancreatitis. Management may consist of fluid support in mild cases to surgical intervention with intensive care therapy in the more severe cases [2]. We present a 23-year-old female patient with an atypical presentation of mild pancreatitis secondary to a ventriculoperitoneal (VP) shunt.

## Case presentation

A 23-year-old female patient presented to the emergency department with a three-day history of epigastric abdominal pain that was associated with nausea. She has a history of congenital hydrocephalus having a VP shunt placed in infancy and revised at age four for obstruction. She is currently breastfeeding, is a non-drinker, non-smoker and has a recent abdominal ultrasound (two months prior) showing no biliary calculi. 

The patient has had multiple recent abdominal surgeries and has subsequently had VP shunt-related complications. She had a recent emergent caesarean section for foetal distress six months prior. She subsequently presented to the emergency department with a painful Richter’s hernia with an abdominal ultrasound showing the tip of the VP shunt tubing present within the hernia with extraperitoneal fluid tracking from this region into the lower anterior abdominal wall. This was managed with a laparoscopic reduction, washout and repair of the Richter’s hernia. A week later she re-presented with severe headaches, photophobia, nausea and vomiting with evidence of VP shunt obstruction. A stereotactic revision of the VP shunt was performed by neurosurgery. She had a right parietal skin incision adjacent to the valve catheter which confirmed the VP shunt obstruction. As the distal catheter was being removed it snapped and a decision was made to leave the remaining portion in situ with the tip draining into the lesser sac. A left subcostal incision was then made in a mini-laparotomy fashion to insert a new distal catheter which was tunnelled and secured to the existing valve before being placed in the general peritoneal cavity. She was subsequently discharged two days later and followed up in the outpatient clinic. 

She presented acutely two months later. She was normotensive, tachycardic with a pulse rate of 106 beats per minute, respiratory rate of 20 breaths per minute and temperature of 37.9 degrees Celsius. She was orientated with a Glasgow Coma Scale 15 with no signs of VP obstruction. She had a soft abdomen but was guarding and tender in the epigastric and right upper quadrant. Her previous incisions had healed and there was no evidence of cellulitis. 

**Figure 1:**
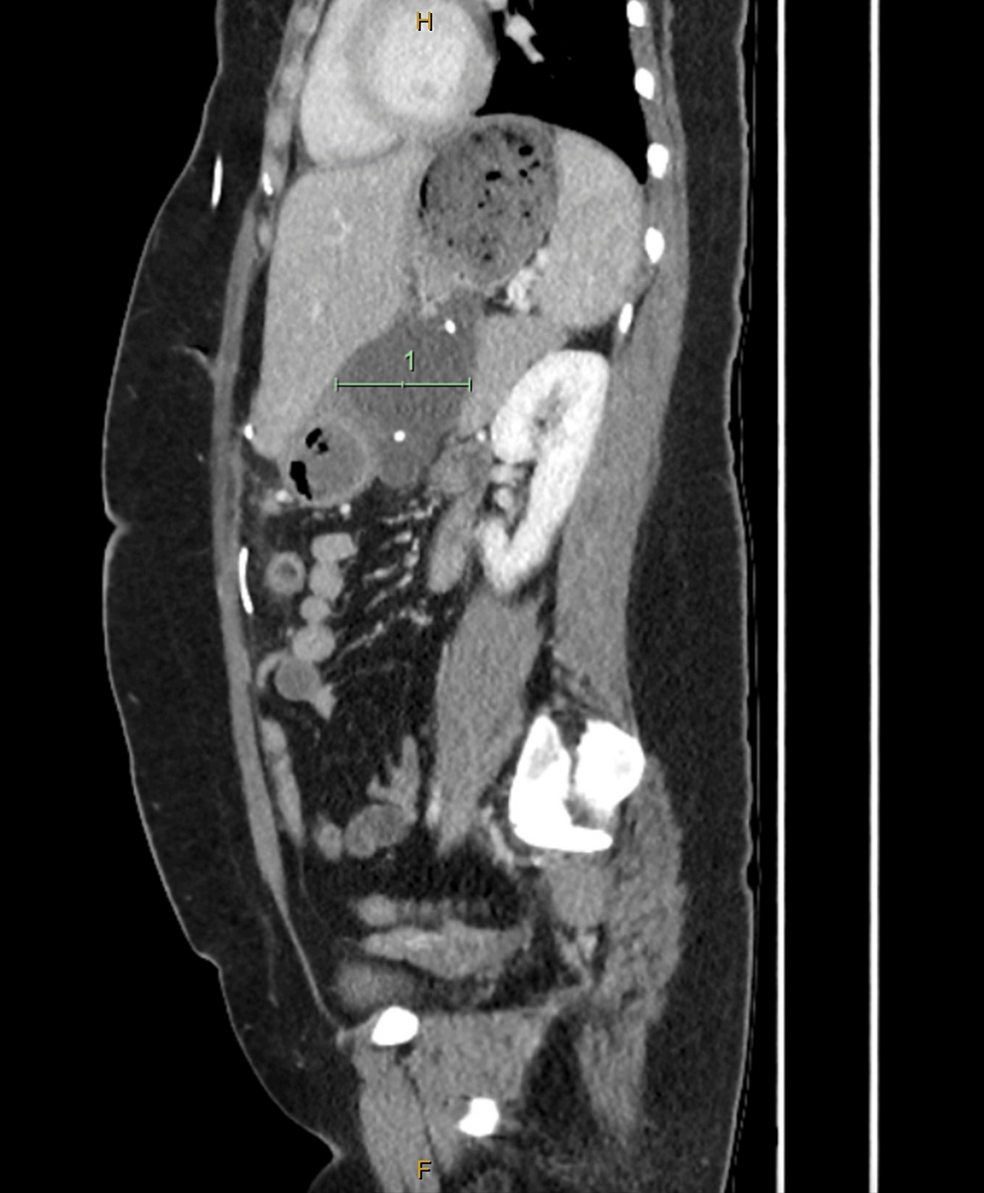
CT sagittal view showing collection within the lesser sac

**Figure 2:**
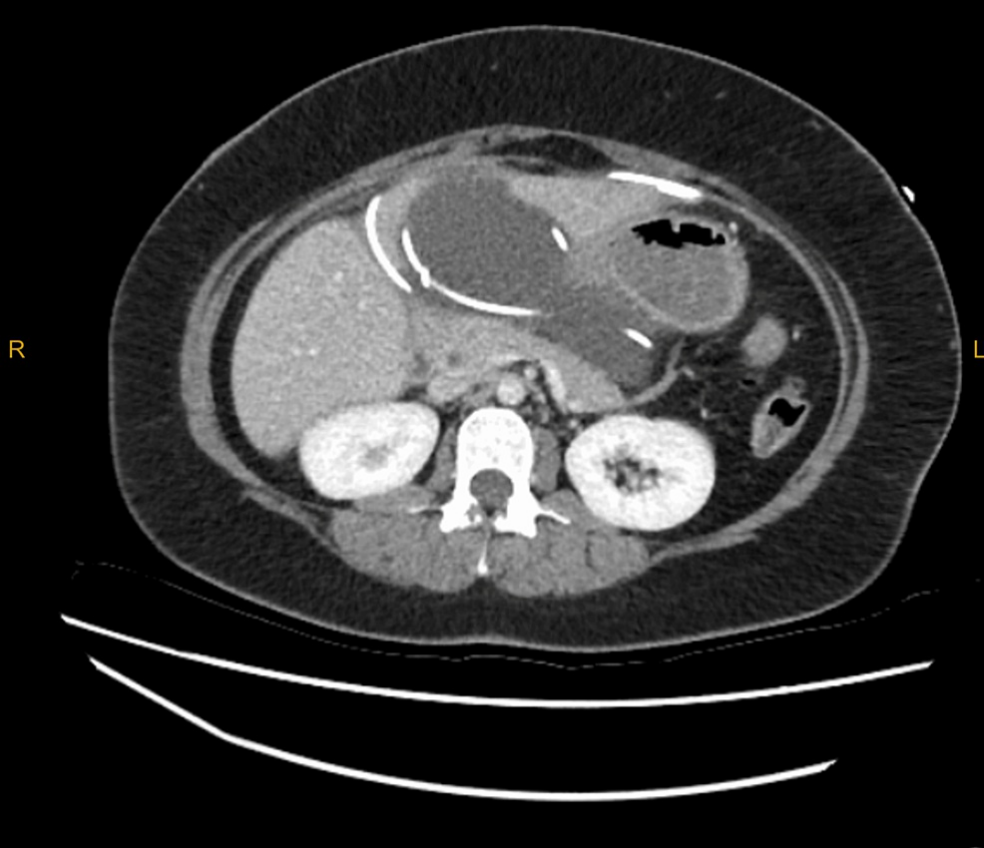
CT abdomen showing catheter tips within the collection

Investigations showed a lipase 3030 (10-60U/L), CRP 157 (<5mg/L) and normal white cell count and liver function tests. CT abdomen showed a walled-off collection measuring approximately 136 mm x 108 mm x 56 mm containing the tips of both VP shunts. There was also some fat stranding neighbouring the pancreas parenchyma and a small volume of free fluid in the right iliac fossa, as seen in Figures 1, 2.

**Figure 3:**
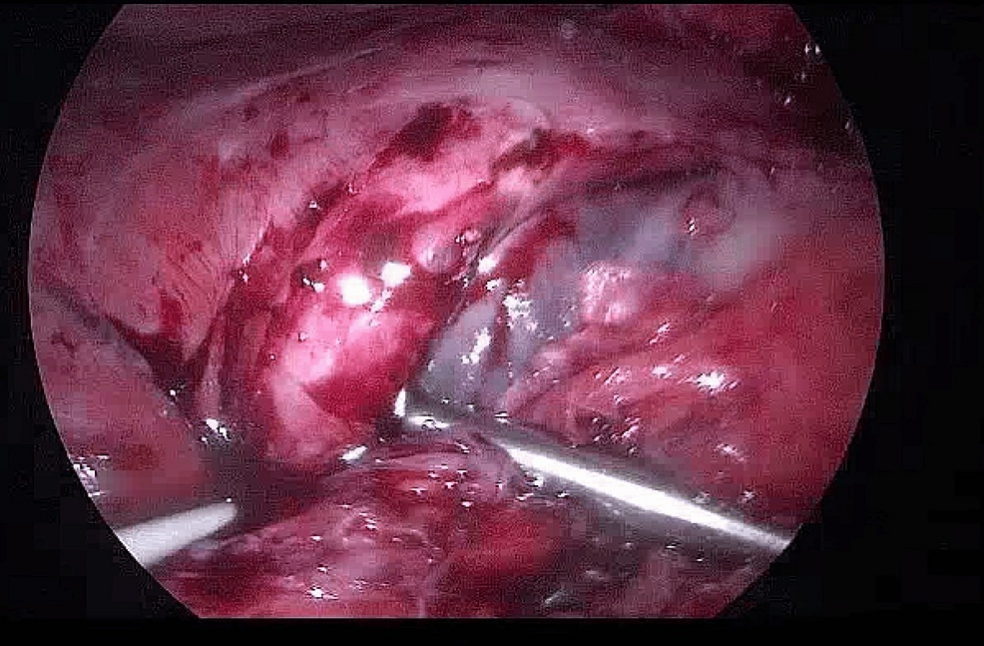
Laparoscopic image of lesser sac collection

**Figure 4:**
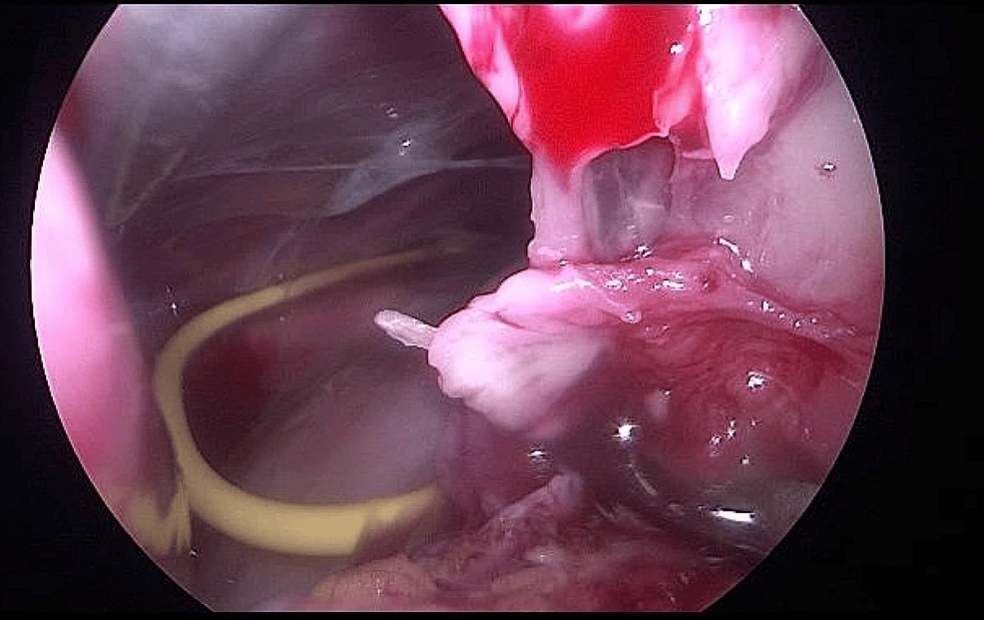
Laparoscopic image showing yellow catheter (new catheter) within the collection following aspiration

She was managed by general surgery and neurosurgery with emergent laparoscopy. Findings included significant abdominal wall adhesions, and a bulging clear collection in the lesser sac where the new tip (yellow) and old tip (white) were found, as seen in Figures 3, 4.

The old tip was surrounded by fibrin and obstructing the foramen of Winslow. The new tip appeared to have migrated into the lesser sac via the omentum and was sitting curled up in the lesser sac. It was externalised due to concerns of infection and the old tip was removed. The fluid was sent for lipase, CSF biochemistry as well as microscopy, culture and sensitivity. The infectious disease team was consulted and she was commenced on cephazolin whilst awaiting further biochemistry. The fluid culture showed no organisms and the CSF shows protein <0.1 (15-40mg/dL), glucose 3.8 (50-80mg/dL), leucocytes 5 (0-5), erythrocytes 5000 (0) and lipase of 75. It was concluded the fluid was CSF, and *Corynebacterium* sensitive to vancomycin was cultured from the old tip.

The VP shunt was internalised on day 8. This was a combined procedure as laparoscopic-assisted placement of the distal catheter. There were upper abdominal omental adhesions and fat saponification secondary to pancreatitis not seen at the initial laparoscopy. This was thought to be the sequelae of the underlying pancreatitis. The VP shunt tip was placed in the right lower quadrant. She was discharged with outpatient follow up which was unremarkable. 

## Discussion

VP shunts have become the standard in the management of hydrocephalus [3,4]. A shunt consists of a ventricular catheter that is connected to a valve and then connected to a distal catheter. The use of the peritoneal cavity as the location for CSF absorption was introduced by Kaush in 1908 [4,5]. Peritoneal placement is thought to be associated with reduced complications compared with ventriculoatrial and ventriculopleural shunts. The peritoneal approach has been described both as a mini-laparotomy or laparoscopic-assisted [6,7]. Pancreatitis is a rare complication of VP shunts and may be associated with the location of the distal catheter [3-5].

Methods of surgical technique described include using the falciform ligament to suspend the catheter directing it to the suprahepatic space or placing it in the general peritoneal cavity [6-8]. The most common complications described include malposition, infection, visceral perforation, shunt erosion of the skin, VP obstruction and pseudocyst formation [3-8]. Utilising the falciform technique has been shown to reduce the risk of malposition compared with general peritoneal placement [8]. 

In this case, there were two distal catheters in situ, both located in the lesser sac increasing the risk of complication. We hypothesise that the new catheter migrating into the lesser sac resulted in pseudocyst formation abutting the body of the pancreas causing trauma and pressure. Leading to inflammation of the pancreas and presentation of acute pancreatitis. The old distal catheter tip may have contributed to this process by trauma to the pancreas after being snapped in the removal process and obstructing the foramen of Winslow with the catheter. 

At the time of the presentation, the radiological findings raised concern about an infectious collection. There was concern this was from the VP shunt rather than the pancreas. The literature supports the externalisation of the shunt in shunt-related intra-abdominal infections. However, there is much debate with regard to the externalisation of non-shunt-related abdominal or pelvic infections [9]. Some publications have stated that there was no statistical evidence of a reduction in chronic peritonitis if the shunt remained in situ. Given the fluid culture had shown no organisms it is important to assess this at the time of laparoscopy to potentially avoid a further operation. 

## Conclusions

The location of the distal VP catheter needs to be considered when placing a VP shunt for hydrocephalus. Pancreatitis is a complication if the VP shunt catheter migrates as demonstrated by this case. Management is dependent on the symptoms, presence of infection and severity of the pancreatitis. The anatomical consideration of the course of the intra abdominal portion of the VP shunt should be considered. This case also demonstrates the collegial nature of collaborative care between two surgical specialities to improve patient outcomes successfully. Adoption of laparoscopic assisted placement of the distal catheter may be beneficial and this should be considered when assessing the patient prior to the initial VP shunt placement or revision surgery. 
